# Downregulation of *SFRP1* is a protumorigenic event in hepatoblastoma and correlates with beta-catenin mutations

**DOI:** 10.1007/s00432-020-03182-1

**Published:** 2020-03-18

**Authors:** Ivonne Regel, Melanie Eichenmüller, Ujjwal Mukund Mahajan, Beate Hagl, Simone Benitz, Beate Häberle, Christian Vokuhl, Dietrich von Schweinitz, Roland Kappler

**Affiliations:** 1 Department of Medicine II, University Hospital, LMU Munich, Munich, Germany; 2 Department of Pediatric Surgery, Dr. von Hauner Children’s Hospital, Ludwig-Maximilians-University Munich, LMU Munich, Lindwurmstr. 2a, 80337 Munich, Germany; 3 Chair and Institute of Environmental Medicine, UNIKA-T, Technical University of Munich and Helmholtz Zentrum Munich, Munich/Augsburg, Munich, Germany; 4 Department of Molecular and Integrative Physiology, University of Michigan, Ann Arbor, MI USA; 5 Institute of Pathology, University Hospital Bonn, Bonn, Germany

**Keywords:** Pediatric liver cancer, Hepatoblastoma, Hepatocellular carcinoma, Epigenetics, DNA methylation, WNT signaling, SFRP1, DKK1, WIF1, APC

## Abstract

**Background:**

Hepatoblastoma (HB) and pediatric hepatocellular carcinoma (HCC) are the most common malignant liver tumors in childhood. Both tumor types exhibit genetic and epigenetic alterations in the WNT/β-catenin signaling pathway, which is a key regulator of liver progenitor cells in embryonic development. The tumors demonstrate a high rate of β-catenin mutations and gene expression changes of several WNT antagonists. However, the role of the WNT inhibitory factor secreted frizzled-related protein 1 (SFRP1) has not been addressed in pediatric liver cancer so far.

**Results:**

In our study, we investigated the gene expression level, DNA methylation status and functional relevance of SFRP1 in HB cell lines and in pediatric liver tumor patient samples. *SFRP1* was downregulated due to DNA promoter methylation in all tested HB cell lines. Overexpression of *SFRP1* in HB cell lines diminished tumor cell proliferation, colony formation and migration potential. In addition, the SFRP1-expressing HB cell lines showed reduced WNT/β-catenin signaling pathway activity and decreased expression of WNT target genes. To evaluate the utility of SFRP1 as a biomarker in pediatric liver cancer, we determined the gene expression level and DNA methylation status of *SFRP1* in 45 pediatric liver tumor patient samples. The correlation analysis of different clinical parameters and tumor characteristics revealed a significant correlation of reduced *SFRP1* expression with the presence of mutant β-catenin. The methylation status of *SFRP1* was furthermore associated to a pediatric liver tumor type with HCC-like characteristics, *TERT* mutations and an older age at diagnosis.

**Conclusion:**

Altogether, our data demonstrate that the epigenetic suppression of the WNT/β-catenin antagonist *SFRP1* has an important impact on the malignant behavior of HB cells. Although *SFRP1* methylation is a common event in HCC-like pediatric liver tumors, its potential as a prognostic or diagnostic biomarker needs to be further investigated.

**Electronic supplementary material:**

The online version of this article (10.1007/s00432-020-03182-1) contains supplementary material, which is available to authorized users.

## Background

Hepatoblastoma (HB) is the most common malignant liver tumor in children under the age of 4 years and its incidence has increased over the last decades (Kremer et al. 2014). Hepatocellular carcinoma (HCC) has also been described in the pediatric population, but the tumor occurs more rarely than HB and predominantly manifest in older children or young adults (Ng and Mogul 2018). Although the survival rates of pediatric HB and HCC have improved up to 80% over the last decades, due to advanced surgical techniques and chemotherapeutic treatments, there are still HB patient subgroups with a dismal prognosis (Tulla et al. 2015; Khanna and Verma 2018; Czauderna and Garnier 2018). Particularly, children presenting with non-resectable tumors, chemotherapy resistance, or metastasis show reduced survival rates (Czauderna et al. 2016). Consequently, a more profound understanding of the biology of HB will help to uncover mechanisms promoting cancer development and progression.

HBs often exhibit an epithelial or mixed morphological subtype and show two distinct transcriptomic profiles, which are classified as 16-gene signature cluster C1 or C2 (Perugorria et al. 2019). Although C1 and C2 HBs differ in their tumor characteristics, they exhibit common genetic aberrations affecting the WNT/β-catenin/AXIN signaling (Cairo et al. 2008). The canonical WNT/β-catenin signaling pathway regulates the proliferation, maturation and survival of liver progenitor cells in embryogenesis. Notably, the pathway is often constitutively activated in HB and HCC development (Russell and Monga 2018). Stabilizing mutations in the β-catenin (*CTNNB1*) gene occur with a frequency of up to 37% in HCC and 60–70% in HB and as a consequence, β-catenin accumulates in the nucleus and regulates the expression of target genes (Zucman-Rossi et al. 2015; Bell et al. 2017). Further genetic alterations involve loss-of-function mutations in the APC regulator of WNT signaling pathway (*APC*), *AXIN1* and *AXIN2* genes, which prevents a proteasomal degradation of β-catenin (Perugorria et al. 2019). Moreover, it was previously shown that the dickkopf WNT signaling pathway inhibitor 1 (*DKK1*) is upregulated in HB patient samples, which might represent negative feedback mechanisms (Wirths et al. 2003). On the contrary, among the known WNT antagonists, the secreted frizzled-related protein 1 (SFRP1) is often downregulated in various cancer entities, which indicates that SFRP1 has tumor-suppressive functions (Vincent and Postovit 2017). Under physiological conditions, SFRP1 inactivates the canonical and non-canonical WNT/β-catenin pathway by directly binding to WNT proteins or the frizzled receptor (Kawano and Kypta 2003). Thus, a downregulation of *SFRP1* in HCC cells resulted in stimulated WNT signaling activity and increased tumor cell growth (Shih et al. 2007). Importantly, during breast and prostate cancer development *SFRP1* expression is lost due to epigenetic silencing, induced by an enrichment of DNA methylation in the promoter region (Lodygin et al. 2005; Lo et al. 2006). Notably, promoter methylation of *SFRP1* was also identified as a common event in adult HCC (Huang et al. 2007; Shih et al. 2006). Since SFRP1 suppression contributes to elevated WNT/β-catenin signaling, which is a known characteristic of HB and HCC, we were highly interested in the functional role of SFRP1 in pediatric liver cancers. Thus, we investigated the *SFRP1* DNA methylation status and gene expression levels in HB cell lines and primary pediatric liver tumor samples. Overexpression of *SFRP1* in HB cell lines resulted in an inhibition of tumor cell growth, colony formation and migration and a decrease in WNT/β-catenin signaling activity. Moreover, *SFRP1* promoter methylation and transcriptional silencing was identified in a subset of primary pediatric liver tumors. Our findings indicate that the epigenetic suppression of *SFRP1* represents an alternative mechanism for enhancing WNT/β-catenin signaling in the development of pediatric liver cancer, particularly in children diagnosed at older ages.

## Methods

### Patients

Liver tumor specimens of 45 patients and matching normal liver tissue from seven patients (N110, N146, N198, N175, N227, N253, N612) were obtained from pediatric patients undergoing surgical resection in the Department of Pediatric Surgery, University Hospital, LMU Munich, Germany. Each patient gave written informed consent and the study protocol was approved by the Committee of Ethics, LMU Munich. Clinicopathological parameters and experimental data of all patient samples are listed in the Supplementary Table 1. Experimental data of *SFRP1* expression were categorized into low (< 1) and high (> 1) and correlated to different clinicopathological parameters. A similar correlation analysis was performed for the *SFRP1* methylation status (*M* methylated, *U* unmethylated).

### Cell culture and DNA methylation inhibitor treatment

The hepatoblastoma cell lines HuH-6 (RRID:CVCL_4381), HepT1 (RRID:CVCL_G003), Hep-T3 (RRID:CVCL_G004), and HepG2 (RRID:CVCL_0027) were cultured in RPMI 1640 growth media (Gibco, Thermo Fischer Scientific, Germany), supplemented with 10% fetal calf serum, 100 U/mL penicillin, and 100 μg/mL streptomycin, at 37 °C in a humidified chamber with a saturated atmosphere containing 5% CO_2_. Cells were passaged at a confluency of 80–90% with 0.05% trypsin (*v*/*v*) and 0.2% EDTA (*w*/*v*) (Sigma-Aldrich, Germany) in Dulbecco’s phosphate-buffered saline (PBS). For gene expression and methylation analysis Huh-6 and Hep-T3 cells were treated with 0.5 µM 5-aza-2′-deoxycytidine (5-aza; Sigma-Aldrich, Germany), HepT1 and HepG2 cells with 1.25 µM 5-aza or solvent for 3 and 5 days.

### Cell transfection

HuH-6, HepT1 and HepG2 cells (5 × 10^5^ cells/six-well plate) were transfected with 1 μg DNA of the pcDNA3.1/V5-HisA control vector (#V81020, Thermo Fischer Scientific, Germany) or the pcDNA3.1-SFRP1 (pSFRP1) expression vector containing full-length *SFRP1* cDNA (Fukui et al. 2005) using FuGene 6 transfection reagent (Roche Diagnostics, Germany) according to the manufacturer’s protocol. For stable transfection, cells were incubated in selection media 24 h after transfection containing 200 µg/ml G418 (Sigma-Aldrich, Germany). Two weeks after G418 selection, resistant colonies were picked and cultured under standard medium conditions.

### Cell viability assay

2000 stably or transiently transfected HuH-6, HepT1 and HepG2 cells were seeded in a 96-well plates in RPMI 1640 growth media and cell proliferation was measured at the indicated time points using the Cell Proliferation Kit I (Roche Diagnostics) according to the manufacturer’s protocol. The absorbance of the colorimetric reaction was quantified on the GENios reader (Tecan, Switzerland) by measuring at a wavelength of 595 nm. Cell growth was normalized to the zero hour time points.

### Colony formation assay

5000 stably transfected HuH-6 and HepG2 cells were seeded in a six-well plate. Cells grew for 10 days in RPMI 1640 growth media. After methanol fixation, cells were stained with 0.05% crystal violet in 20% methanol and washed with tap water. Colonies were counted and are represented as number of colonies per well.

### Cell migration assay

Stably transfected HuH-6 cells were seeded into six-well plates and grown as confluent monolayer. A wound of approximately 1 mm was inflicted to the cell monolayer using a pipette tip. The cells were washed twice with PBS to remove detached cells and incubated for additional 72 h in 1% FCS starved RPMI 1640 growth media to diminish cell proliferation. Images were taken at 0, 24, 48 and 72 h after scratching and the wound widths were measured and quantified with ImageJ (Rasband, W.S., ImageJ, US National Institutes of Health, Bethesda, USA). Cell migration was normalized to the zero hour time points.

### TOP/FOP assay

To measure the activity of the canonical WNT/β-catenin pathway, we used the TOP/FOP-flash promoter assay (Millipore, Germany). 1 × 10^5^ stably transfected HuH-6, HepT1 and HepG2 cells were seeded in a 12-well plate and co-transfected with FuGene 6 transfection reagent (Roche Diagnostics) using the following plasmids: pTOP (Firefly-Luciferase reporter plasmid containing several TCF binding sites) or pFOP (Firefly-Luciferase reporter plasmid containing mutated TCF binding sites) together with pRL-TK (Renilla-Luciferase control plasmid to normalize transfection efficiency). The luciferase activity was measured with the Dual-Glo™ Luciferase Assay System (Promega, Germany) 48 h after transfection according to the manufacturer’s protocol on the GENios microplate reader (Tecan).

### Immunofluorescence staining

Staining was performed according to standard protocol using β-catenin (D10A8) antibody (#8480, cell signaling, USA). Negative control was performed with secondary antibody only (data not shown).

### Immunoblot analysis

Immunoblot analysis was performed according to the manufacturer’s protocol, with the exception that blocking and first antibody (SFRP1 (D5A7) #3534, cell signaling, USA; GAPDH, H86504M, Meridian Life Science, USA) incubation was done in 5% BSA/TBS-T buffer. Secondary antibody was incubated in 5% milk/TBS-T.

### Quantitative real-time PCR (qRT-PCR)

Total RNA was extracted using TriReagent (Sigma-Aldrich, Germany) according to the manufacturer’s protocol. Two micrograms of RNA was transcribed into cDNA using random hexamer primer and SuperScript™ II Reverse Transcriptase (Thermo Fischer Scientific, Germany). Quantitative RT-PCR was performed in doublets using iTaq-SYBR Green-Supermix (Bio-Rad, Germany) and the Master cycler ep gradient (Eppendorf, Germany) as previously described (Eichenmuller et al. 2009). Gene expression primer are provided in Supplementary Table 2. Expression levels were normalized to the housekeeping gene TATA-box binding protein *(TBP)* and the fold change was calculated according to the ΔΔCt method in relation to the expression level of normal liver tissue. An expression level < 1 represents a low and > 1 a high expression.

### Methylation analyses

Genomic DNA was isolated by phenol and chloroform extraction following standard procedures. As a positive control for methylated DNA, genomic DNA of a healthy donor was artificially methylated using the CpG methyltransferase M. SssI (Thermo Fischer Scientific, Germany) according to the manufacturer's instructions. As a negative control for unmethylated DNA, HuH-6 cells were treated with 0.5 μM 5-aza-2′-deoxycytidine (5-aza; Sigma-Aldrich, Germany) for 72 h and genomic DNA was isolated. Genomic DNA was bisulfite-treated using the EpiTect® Bisulfite Kit (Qiagen, Hilden, Germany) according to the manufacturer’s protocol. The methylation status of *APC*, *DKK1*, *SFRP1* and *WIF1* was analyzed by methylation-specific-PCR (MSP). MSP primer are provided in Supplementary Table 2 (Aguilera et al. 2006; Esteller et al. 2000). MSP primer design and PCR conditions were previously described (Eichenmuller et al. 2009). PCR products were visualized on a 1.5% agarose gel.

### Chromatin immunoprecipitation

HuH-6, HepT1 and HepG2 cells were treated with 1.5 µM 5-aza, 1.5 µM 5-aza plus 0.5 µM Vorinostat (SAHA) (SML0061, Sigma-Aldrich, Germany) or dissolvent (DMSO, 1:1000) for 3 days. Chromatin immunoprecipitation was performed as previously described (Benitz et al. 2019). Here, we used the following antibodies: anti-H3K27ac (Acetyl-Histone H3 (Lys27) (D5E4), #8173, Cell Signaling, USA), or IgG control (sc-2027, Santa Cruz Biotechnology, USA). Pull-down was done with Protein A agarose/salmon sperm DNA (Merck Millipore, Germany). After reverse crosslinking, DNA was purified with the Qiaquick® PCR Purification Kit (Qiagen) and two genomic areas around transcriptional start side of *SFRP1* were amplified and quantified by qRT-PCR. Analysis of the *EPCAM* promoter was included as quality control to ensure specific enrichment of the activating histone modifications. Primer sequences are listed in Supplementary Table 2. Sample values were calculated according to the percent input method.

### Statistical analysis

Data are presented as mean ± SEM. Statistical significance was determined by two-tailed, unpaired Student’s *t *test, one-way ANOVA with Dunnett’s multiple comparisons test or Chi-square test as indicated in the figure legends using GraphPad Prism 8 software (GraphPad Software Inc.) or R 2.1.0 (https://cran.r-project.org/src/base/R-2/R-2.1.0.tar.gz) and R-studio Version 1.1.442. A two-sided significance of *p* < 0.05 was used throughout, *p < 0.05, **p < 0.01, ***p < 0.001, ****p < 0.0001.

## Results

### Promoter methylation causes *SFRP1* silencing in human HB cell lines

**Fig. 1:**
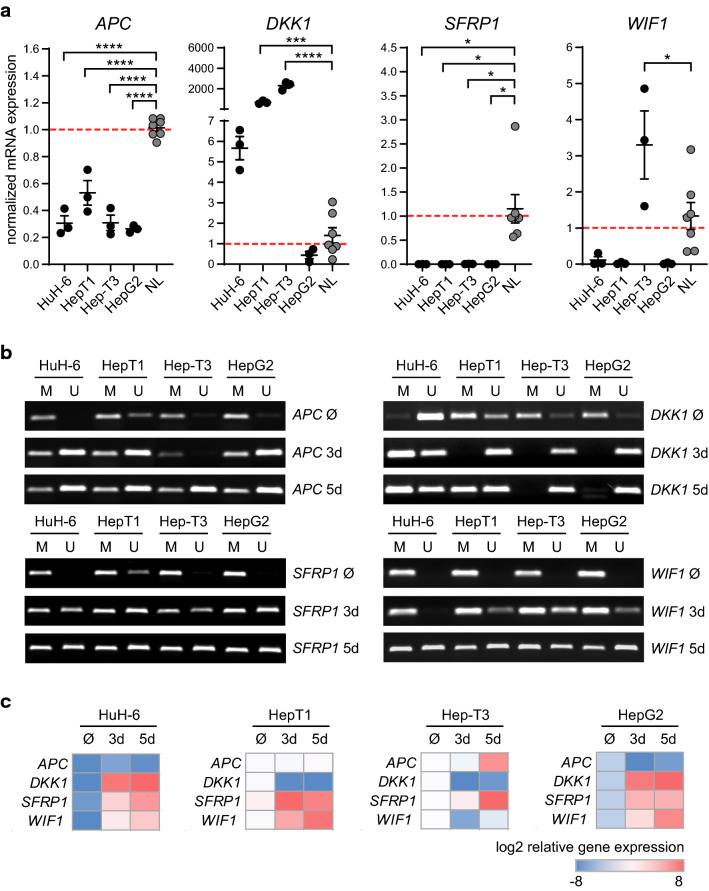
Promoter methylation causes *SFRP1* silencing in human HB cell lines. **a** mRNA expression of *APC*, *DKK1*, *SFRP1* and *WIF1* in HuH-6, HepT1, Hep-T3, and HepG2 and normal liver (NL, *n* = 7) was determined by qRT-PCR (*n* = 3) and calculated as normalized mRNA expression (fold change) to normal liver controls. **b** DNA methylation status (*M* methylated, *U* unmethylated) of *APC*, *DKK1*, *SFRP1* and *WIF1* promoter regions in HuH-6, HepT1, Hep-T3, and HepG2 after solvent (Ø), 3 days (3d) and 5 days (5d) 5-aza treatment was conducted by MSP. **c** mRNA expression of *APC*, *DKK1*, *SFRP1* and *WIF1* in HuH-6, HepT1, Hep-T3, and HepG2 after solvent (Ø), 3 days (3d) and 5 days (5d) 5-aza treatment (*n* = 3) was determined by qRT-PCR and summarized as log_2_ relative gene expression in heatmaps. All data are represented as mean ± SEM; *p* values were calculated by one-way ANOVA with Dunnett’s multiple comparisons test; *p < 0.05, ***p < 0.001, ****p < 0.0001

Although the WNT/β-catenin signaling pathway is often constitutively activated in liver cancer, due to stabilizing β-catenin mutations, previous data indicate that an additional suppression of the WNT antagonist, particularly of SFRP1, through epigenetic mechanisms is an important event in cancer formation (Kaur et al. 2012). To analyze the gene expression status of the known WNT antagonists *APC*, *DKK1*, *SFRP1* and *WIF1* in HB, we selected the HB cell lines HuH-6, HepT1, Hep-T3 and HepG2 and compared the expression levels to normal pediatric liver tissue (NL) (Fig. 1a). Here, *APC* and *SFRP1* were downregulated in all four HB cell lines. In contrast, *DKK1* and *WIF1* showed a heterogeneous expression pattern, with an increased expression of *DKK1* in HuH-6, HepT1 and Hep-T3 and of *WIF1* in Hep-T3 cells (Fig. 1a). Next, we investigated whether the gene expression of *APC*, *DKK1*, *SFRP1* and *WIF1* is epigenetically controlled by DNA promoter methylation. For this, we performed methylation-specific-PCR (MSP) after bisulfite treatment of DNA isolated from HB cell lines and identified that all four HB cell lines demonstrated a strong DNA promoter methylation of the *APC*, *DKK1*, *SFRP1* and *WIF1* genes, with an exception for *DKK1*, which was unmethylated in HuH-6 cells (Fig. 1b). Treatment with the DNA methylation inhibitor 5-aza-2´-deoxycytidine (5-aza) for 3 and 5 days revealed a strong DNA demethylation of the analyzed promoter areas in all cell lines (Fig. 1b). However, 5-aza treatment did not affect *APC* expression. *DKK1* and *WIF1*, which show originally a high expression in HepT1 and/or Hep-T3 cells, were unexpectedly downregulated after 5-aza treatment (Fig. 1c). Interestingly, only the demethylation of the *SFRP1* promoter was associated with a consistent restoration of gene expression in all four HB cell lines (Fig. 1c). Immunoblot analysis of 5-aza-treated HuH6, HepT1 and HepG2 cells revealed increased SFRP1 expression after 3 days (Supplementary Fig. 1a). Moreover, we analyzed if other epigenetic mechanisms, such as histone acetylation, regulate *SFRP1* gene expression. The 5-aza treatment of HuH-6, HepT1 and HepG2 cells alone or in combination with an HDAC inhibitor (SAHA) did not result in increased histone acetylation levels at two regulatory *SFRP1* genome sites, as it could be shown for *EPCAM*, which was included as positive control (Supplementary Fig. 1b). In conclusion, our data suggest that solely *SFRP1* promoter hypermethylation is associated with a transcriptional silencing in HB tumor cell lines.

### Restored *SFRP1* expression affects WNT signaling activity and HB tumor cell characteristics

**Fig. 2:**
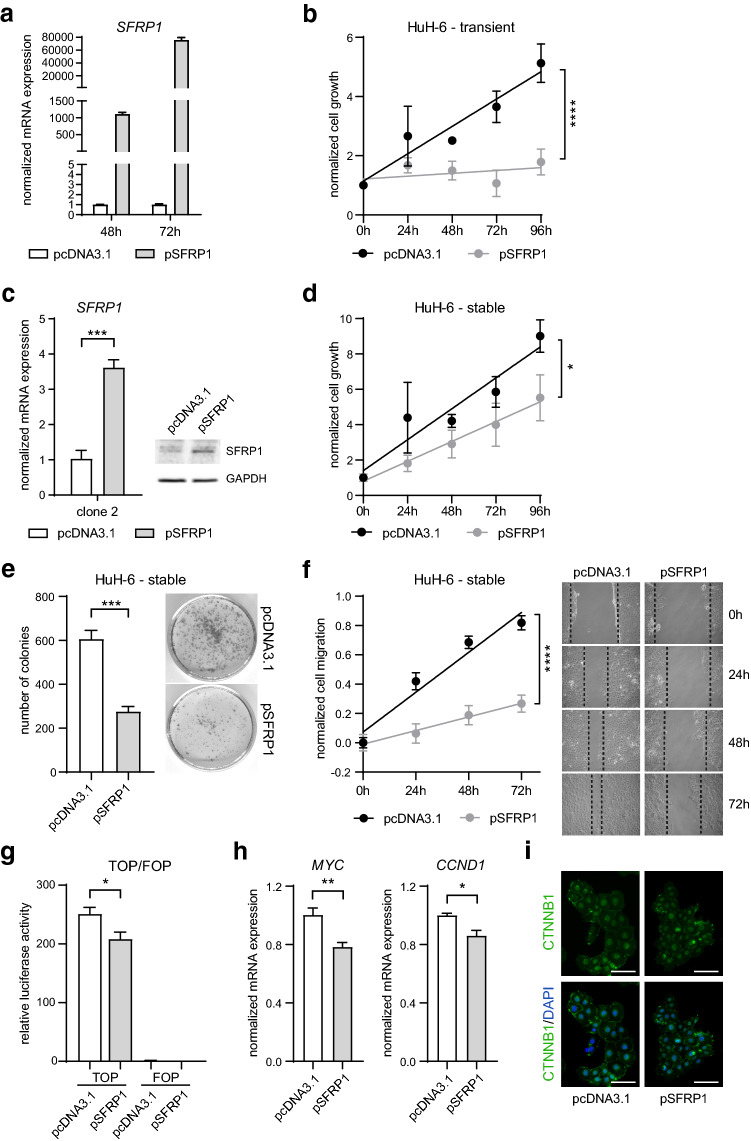
Restored *SFRP1* expression affects WNT signaling activity and HB tumor cell characteristics. **a** mRNA expression of *SFRP1* in transient pcDNA3.1- and pSFRP1-transfected HuH-6 cells after 48 and 72 h was determined by qRT-PCR and calculated as normalized mRNA expression (fold change) to pcDNA3.1 control (*n* = 2). **b** Cell growth of transient pcDNA3.1- and pSFRP1-transfected HuH-6 cells was assessed by MTT assay at indicated time points. Values were normalized to zero hour time point and shown as mean ± SEM (*n* = 2). Slope difference was analyzed by linear regression, ****p < 0.0001. **c** mRNA expression and representative immunoblot image of SFRP1 in stable pcDNA3.1- and pSFRP1-transfected HuH-6 cell clone 2. GAPDH served as loading control in immunoblot analysis (*n* = 2). Gene expression was determined by qRT-PCR and calculated as normalized mRNA expression (fold change) to pcDNA3.1 control (*n* = 3). **d** Cell growth of stable pcDNA3.1- and pSFRP1-transfected HuH-6 cells was assessed by MTT assay at indicated time points. Values were normalized to zero hour time point and shown as mean ± SEM (*n* = 4). Slope difference was analyzed by linear regression, *p < 0.05. **e** Representative pictures and quantification of number of colonies per well of stable pcDNA3.1- and pSFRP1-transfected HuH-6 cells (*n* = 3). **f** Representative pictures and quantification of cell migration at indicated time points were normalized to zero hour time points. Slope difference was analyzed by linear regression, ****p < 0.0001. **g** TOP/FOP reporter plasmid activity was assessed by a relative luciferase activity in stable pcDNA3.1- and pSFRP1-transfected HuH-6 cells 48 h after co-transfection (*n* = 5). **h** mRNA expression of *MYC* and *CCND1* in stable pcDNA3.1- and pSFRP1-transfected HuH-6 cell clone 2 was determined by qRT-PCR and calculated as normalized mRNA expression (fold change) to pcDNA3.1 control (*n* = 4). **i** Immunofluorescence staining of β-catenin (CTNNB1, green) and DAPI nuclear staining (blue) in stable pcDNA3.1- and pSFRP1-transfected HuH-6 cells. Scale bars: 100 µm. All data are represented as mean ± SEM; unless otherwise stated *p* values were calculated by two-tailed, unpaired Student’s *t *test; *p < 0.05, **p < 0.01, ***p < 0.001, ****p < 0.0001

To study the functional relevance of *SFRP1* gene silencing in HB, we assessed tumor cell characteristics after *SFRP1* re-expression. To restore *SFRP1* gene expression, we transiently transfected HuH-6, HepT1 and HepG2 cells with the pcDNA3.1-SFRP1 plasmid (pSFRP1), containing full-length *SFRP1* cDNA, or with the empty vector (pcDNA3.1) as control. Expression levels of *SFRP1* were determined 48 and 72 h after transfection and showed markedly elevated *SFRP1* transcript levels (Fig. 2a, Supplementary Fig. 2a). Notably, transiently pSFRP1-transfected HuH-6, HepT1 and HepG2 cells displayed a reduced growth rate compared to control-transfected cells (Fig. 2b, Supplementary Fig. 2b). To analyze long-term effects, we generated pSFRP1 or pcDNA3.1 stably transfected HuH-6, HepT1 and HepG2 cells. For instance, the HuH-6 cell clone 2 showed a substantial increase in SFRP1 gene and protein expression (Fig. 2c). In line with our preceding results, the stable *SFRP1* expression in HuH-6, HepT1 and HepG2 cells resulted in significantly impaired tumor cell growth (Fig. 2d, Supplementary Fig. 2c) and a strong decrease in colony formation (Fig. 2e, Supplementary Fig. 2d) and migration capacity (Fig. 2f) compared to control cells. To investigate whether the re-expression of *SFRP1* has a direct influence on the activity of the canonical WNT/β-catenin signaling pathway, we performed a TOP/FOP luciferase reporter assay, in which the binding activity of ß-catenin to the TOP reporter plasmids is measured. Strikingly, the stably pSFRP1-transfected HuH-6, HepT1 and HepG2 cells revealed a substantial reduction in the relative luciferase activity, indicating a suppression of the canonical WNT/β-catenin pathway activity (Fig. 2g, Supplementary Fig. 2e). To corroborate these findings, we additionally measured the expression of WNT target genes and observed a significant downregulation of *MYC* and *CCND1* in the stably pSFRP1-transfected HuH-6 cells (Fig. 2h). Interestingly, the level of β-catenin or its cellular localization is not altered in the stably pSFRP1-transfected HuH-6 cells, indicating that *SFRP1* re-expression abolishes the transcriptional activity of β-catenin (Fig. 2i). Altogether, our results demonstrate that a restored SFRP1 expression has tumor-suppressive effects in HB cells by reducing the activity of the canonical WNT/β-catenin pathway.

### *SFRP1* DNA methylation correlates with the tumor type and a late onset of the disease

**Fig. 3:**
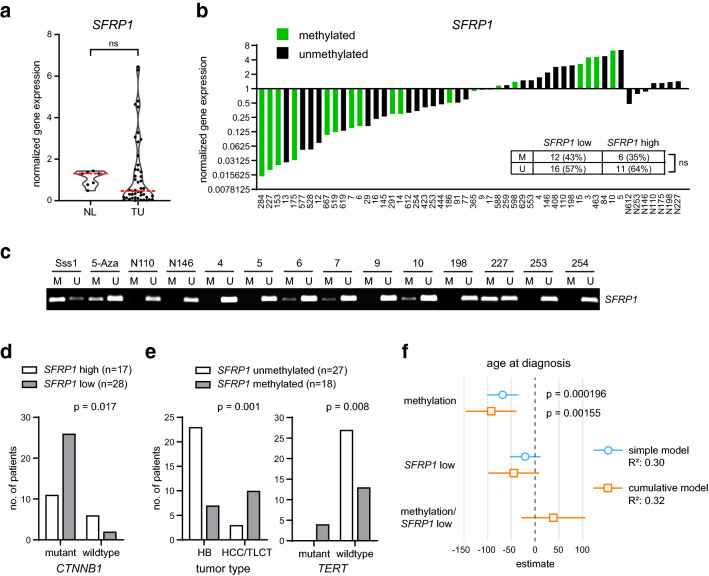
*SFRP1* DNA methylation correlates with the tumor type and late onset of the disease. **a** mRNA expression of *SFRP1* in normal liver (NL) and patient tumor samples (TU) was determined by qRT-PCR and calculated as normalized mRNA expression (fold change) to NL controls. The red line marks the median expression level. **b** For each tumor and normal tissue (*N*—number) sample, the normalized *SFRP1* gene expression and promoter methylation status is shown. The embedded table displays the distribution of methylated (M) and unmethylated (U) *SFRP1* in the *SFRP1* low and high gene expression categories. **c** Representative pictures of the *SFRP1* MSP reaction from selected normal and tumor tissue samples. Sss1-treated DNA serves as methylated positive control, 5-aza-treated DNA as unmethylated control. **d** Illustration of the correlation analysis of *SFRP1* gene expression and β-catenin mutation status, see also Table 1. **e** Illustration of the correlation analysis of *SFRP1* DNA methylation status to the tumor type, considering only HB and HCC/TLCT tumors, and to the *TERT* mutation status, see also Table 2. **f** Forest plot with a simple and cumulative generalized linear regression model considering *SFRP1* methylation status, gene expression category and a combined profile in respect to the age at diagnosis

To uncover the *SFRP1* gene expression and DNA methylation status in primary pediatric liver tumors, we first performed a gene expression analysis on a cohort of 45 patient samples, containing 30 HBs, nine HCCs, 2 transitional liver cell tumors (TLCT) and 2 nested stromal-epithelial liver tumors (NSET) (Supplementary Table 1). Overall, we detected a low *SFRP1* gene expression in 62% (28/45) of cases and the median *SFRP1* expression level was reduced in tumor tissue compared to normal liver, although the difference was not significant (Fig. 3a, b). In addition, we determined the *SFRP1* DNA methylation status for each case with MSP and correlated it to the *SFRP1* gene expression level (Fig. 3b). Notably, 43% of the patient samples with low *SFRP1* expression showed *SFRP1* DNA methylation. Concomitantly, however, 35% of the patient samples with a high *SFRP1* expression showed also *SFRP1* DNA methylation. Hence, the overall correlation between the *SFRP1* DNA methylation and transcriptional status was not significant (Fig. 3b). Representative results of the MSP reactions are illustrated in Fig. 3c. To study the impact of the *SFRP1* gene expression level on clinical outcome, we performed a Chi-square correlation analysis based on a low (< 1) and high (> 1) *SFRP1* expression and various clinicopathological parameters (Table 1). Here, we uncovered that the *SFRP1* expression is significantly associated with the PRETEXT (PRE-Treatment EXTent of tumor) risk classification system (Towbin et al. 2018). Particularly, all patient samples (6/6) with the unfavorable PRETEXT category 4 displayed a low *SFRP1* gene expression (Table 1). Moreover, the transcriptional level of *SFRP1* correlated significantly with the ß-catenin (*CTNNB1)* mutation status. Interestingly, patient samples with mutant ß-catenin demonstrated a reduction in *SFRP1* gene expression, whereas patients with wildtype ß-catenin revealed increased *SFRP1* expression levels (Fig. 3d). Since 18 out of 45 patient samples exhibited *SFRP1* DNA methylation, we performed also a Chi-square correlation analysis based on a methylated (M) and unmethylated (U) *SFRP1* profile (Table 2). The *SFRP1* methylation status was significantly associated with the gender, age at diagnosis, tumor type, differentiation, extrahepatic growth, resection margin and telomerase reverse transcriptase (*TERT*) mutations (Table 2). The distribution of *SFRP1-*methylated and -unmethylated cases in relation to the tumor type clearly showed that most of the HCC/TLCT samples displayed an enrichment of *SFRP1* DNA methylation and that all tumors with a *TERT* mutation were *SFRP1-*methylated (Fig. 3e). Of further note, *SFRP1* DNA methylation as a single parameter was significantly associated with an older age at diagnosis, although a low *SFRP1* expression did not correlate with the age in a simple or cumulative correlation model (Fig. 3f). Overall, our findings revealed that *SFRP1* DNA methylation and transcriptional silencing is a common event in pediatric liver cancer and that *SFRP1* DNA methylation is a preferential characteristic of pediatric liver tumors with HCC-like features, such as hepatocellular histology, advanced age and *TERT* mutations.

**Table 1:** Correlation analysis of *SFRP1* gene expression

*SFRP1* expression	High (*n* = 17)	Low (*n* = 28)	Total (*n* = 45)	*p* value
Gender				0.848
f	8 (47.1%)	14 (50.0%)	22 (48.9%)	
m	9 (52.9%)	14 (50.0%)	23 (51.1%)	
Age at diagnosis in month				0.386
nd	0	1	1	
Mean (SD)	67.867 (72.955)	50.780 (55.905)	57.382 (62.777)	
Range	1.874–199.435	0.000–184.179	0.000–199.435	
Outcome				0.758
DOD	3 (17.6%)	6 (21.4%)	9 (20.0%)	
NED	14 (82.4%)	22 (78.6%)	36 (80.0%)	
Cause of death				0.541
nd	0	1	1	
Progressive	2 (11.8%)	2 (7.4%)	4 (9.1%)	
Recurrence	2 (11.8%)	3 (11.1%)	5 (11.1%)	
Alive	13 (76.5%)	22 (81.5%)	35 (79.5%)	
Tumor type				0.170
HB	10 (58.8%)	20 (71.4%)	30 (66.7%)	
HCC/TLCT	5 (29.4%)	8 (28.6%)	13 (28.9%)	
NSET	2 (11.8%)	0 (0.0%)	2 (4.4%)	
Differentiation				0.388
nd	2	0	2	
Epithelial	6 (40.0%)	17 (60.7%)	23 (53.5%)	
Fibrolamellar	3 (20.0%)	1 (3.6%)	4 (9.3%)	
Well differentiated	0 (0.0%)	2 (7.1%)	2 (4.4%)	
Moderately differentiated	1 (6.7%)	2 (7.1%)	3 (7.0%)	
Mixed	5 (33.3%)	6 (21.4%)	11 (25.6%)	
Component				0.332
na	6 (35.3%)	5 (17.9%)	11 (24.4%)	
E	1 (5.9%)	0 (0.0%)	1 (2.2%)	
E/F	3 (17.6%)	6 (21.4%)	9 (20.0%)	
E > F	1 (5.9%)	1 (3.6%)	2 (4.4%)	
F	2 (11.8%)	4 (14.3%)	6 (13.3%)	
F > E	3 (17.6%)	12 (42.9%)	15 (33.3%)	
Pure OS	1 (5.9%)	0 (0.0%)	1 (2.2%)	
Stage				0.296
nd	2	1	3	
I	1 (6.7%)	6 (22.2%)	7 (16.7%)	
II	0 (0.0%)	1 (3.7%)	1 (2.4%)	
III	9 (60.0%)	9 (33.3%)	18 (42.9%)	
IV	5 (33.3%)	11 (40.7%)	16 (38.1%)	
PRETEXT				**0.025**
nd	3	1	4	
1	2 (13.3%)	0 (0.0%)	2 (4.8%)	
2	3 (20.0%)	11 (40.7%)	14 (33.3%)	
3	9 (60.0%)	10 (37.0%)	19 (45.2%)	
4	0 (0.0%)	6 (22.2%)	6 (14.3%)	
Extrahepatic				0.820
nd	0	2	2	
No	16 (94.1%)	24 (92.3%)	40 (93.0%)	
Yes	1 (5.9%)	2 (7.7%)	3 (7.0%)	
Multifocal				0.666
nd	2	1	3	
No	12 (80.0%)	20 (74.1%)	32 (76.2%)	
Yes	3 (20.0%)	7 (25.9%)	10 (23.8%)	
Metastasis				0.447
nd	0	1	1	
No	12 (70.6%)	16 (59.3%)	28 (63.6%)	
Yes	5 (29.4%)	11 (40.7%)	16 (36.4%)	
Chemotherapy				0.282
No	2 (11.8%)	7 (25.0%)	9 (20.0%)	
Yes	15 (88.2%)	21 (75.0%)	36 (80.0%)	
Resection margin				0.911
Nd	2	1	3	
R0	13 (81.2%)	23 (82.1%)	36 (81.8%)	
R1	2 (12.5%)	4 (14.3%)	6 (13.6%)	
16-gene signature				0.172
na	2 (11.8%)	0 (0.0%)	2 (4.4%)	
C1	9 (52.9%)	18 (64.3%)	27 (60.0%)	
C2	6 (35.3%)	10 (35.7%)	16 (35.6%)	
CTNNB1				**0.017**
Mutant	11 (64.7%)	26 (92.9%)	37 (82.2%)	
Wildtype	6 (35.3%)	2 (7.1%)	8 (17.8%)	
NFE2L2				0.137
nd	3	0	3	
Mutant	0 (0.0%)	4 (14.3%)	4 (9.5%)	
Wildtype	14 (100.0%)	24 (85.7%)	38 (90.5%)	
TERT				0.620
nd	1	0	1	
Mutant	1 (6.2%)	3 (10.7%)	4 (9.1%)	
Wildtype	15 (93.8%)	25 (89.3%)	40 (90.9%)	
*SFRP1* methylation				0.616
M	6 (35.3%)	12 (42.9%)	18 (40.0%)	
U	11 (64.7%)	16 (57.1%)	27 (60.0%)	

Chi-square correlation analysis of normalized low (< 1) and high (> 1) *SFRP1* expression categories to different clinicopathological and experimental parameters

*m* male, *f* female, *DOD* died of disease, *NED* no evidence of disease, *HB* hepatoblastoma, *HCC* hepatocellular carcinoma, *TLCT* transitional liver cell tumor, *NSET* nested stromal-epithelial liver tumor, *E* embryonal, *F* fetal, *pure OS* pure osteoid, *C1 and C2* 16-gene signature cluster C1 and C2 (Cairo et al. 2008), *M* methylated, *U* unmethylated, *nd* no data, *na* not applicable

**Table 2:** Correlation analysis of *SFRP1* promoter methylation

*SFRP1* methylation	M (*n* = 18)	U (*n* = 27)	Total (*n* = 45)	*p* value
Gender				**0.021**
f	5 (27.8%)	17 (63.0%)	22 (48.9%)	
m	13 (72.2%)	10 (37.0%)	23 (51.1%)	
Age at diagnosis in month				** < 0.001**
nd	1	0	1	
Mean (SD)	98.48 (73.09)	31.50 (37.82)	57.38 (62.78)	
Range	7.37–199.44	0.00–156.33	0.00–199.44	
Outcome				0.287
DOD	5 (27.8%)	4 (14.8%)	9 (20.0%)	
NED	13 (72.2%)	23 (85.2%)	36 (80.0%)	
Cause of death				0.512
nd	1	0	1	
Progressive	2 (11.8%)	2 (7.4%)	4 (9.1%)	
Recurrence	3 (16.6%)	2 (7.4%)	5 (11.1%)	
Alive	12 (70.6%)	23 (85.2%)	35 (79.5%)	
Tumor type				**0.004**
HB	7 (38.9%)	23 (85.2%)	30 (66.7%)	
HCC/TLCT	10 (55.6%)	3 (11.1%)	13 (28.9%)	
NSET	1 (5.6%)	1 (3.7%)	2 (4.4%)	
Differentiation				**0.042**
nd	1	1	2	
Epithelial	8 (47.1%)	15 (57.7%)	23 (53.5%)	
Fibrolamellar	4 (23.5%)	0 (0.0%)	4 (9.3%)	
Well differentiated	1 (5.9%)	1 (3.8%)	2 (4.4%)	
Moderately differentiated	2 (11.8%)	1 (3.8%)	3 (7.0%)	
Mixed	2 (11.8%)	9 (34.6%)	11 (25.6%)	
Component				0.260
na	8 (44.4%)	3 (11.1%)	11 (24.4%)	
E	0 (0.0%)	1 (3.7%)	1 (2.2%)	
E/F	3 (16.7%)	6 (22.2%)	9 (20.0%)	
E > F	1 (5.6%)	1 (3.7%)	2 (4.4%)	
F	2 (11.1%)	4 (14.8%)	6 (13.3%)	
F > E	4 (22.2%)	11 (40.7%)	15 (33.3%)	
pure OS	0 (0.0%)	1 (3.7%)	1 (2.2%)	
Stage				0.078
nd	1	2	3	
I	5 (29.4%)	2 (8.0%)	7 (16.7%)	
II	1 (5.9%)	0 (0.0%)	1 (2.4%)	
III	4 (23.5%)	14 (56.0%)	18 (42.9%)	
IV	7 (41.2%)	9 (36.0%)	16 (38.1%)	
PRETEXT				0.552
nd	4	0	4	
1	1 (6.7%)	1 (3.7%)	2 (4.8%)	
2	6 (40.0%)	8 (29.6%)	14 (33.3%)	
3	5 (33.3%)	14 (51.9%)	19 (45.2%)	
4	2 (13.3%)	4 (14.8%)	6 (14.3%)	
Extrahepatic				**0.020**
nd	2	0	2	
No	13 (81.2%)	27 (100.0%)	40 (93.0%)	
Yes	3 (18.8%)	0 (0.0%)	3 (7.0%)	
Multifocal				0.280
nd	3	0	3	
No	10 (66.7%)	22 (81.5%)	32 (76.2%)	
Yes	5 (33.3%)	5 (18.5%)	10 (23.8%)	
Metastasis				0.598
nd	1	0	1	
No	10 (58.8%)	18 (66.7%)	28 (63.6%)	
Yes	7 (41.2%)	9 (33.3%)	16 (36.4%)	
Chemotherapy				0.761
No	4 (22.2%)	5 (18.5%)	9 (20.0%)	
Yes	14 (77.8%)	22 (81.5%)	36 (80.0%)	
Resection margin				**0.046**
nd	3	0	3	
R0	11 (64.7%)	25 (92.6%)	36 (81.8%)	
R1	4 (23.5%)	2 (7.4%)	6 (13.6%)	
16-gene signature				0.871
na	1 (5.6%)	1 (3.7%)	2 (4.4%)	
C1	10 (55.6%)	17 (63.0%)	27 (60.0%)	
C2	7 (38.9%)	9 (33.3%)	16 (35.6%)	
CTNNB1				0.340
Mutant	16 (88.9%)	21 (77.8%)	37 (82.2%)	
Wildtype	2 (11.1%)	6 (22.2%)	8 (17.8%)	
NFE2L2				0.571
nd	2	1	3	
Mutant	1 (6.2%)	3 (11.5%)	4 (9.5%)	
Wildtype	15 (93.8%)	23 (88.5%)	38 (90.5%)	
TERT				**0.008**
nd	1	0	1	
Mutant	4 (23.5%)	0 (0.0%)	4 (9.1%)	
Wildtype	13 (76.5%)	27 (100.0%)	40 (90.9%)	
*SFRP1* expression				0.908
Mean (SD)	1.327 (1.956)	1.265 (1.575)	1.290 (1.716)	
Range	0.015–6.287	0.029–6.450	0.015–6.450	
*SFRP1* exp. category				0.616
High	6 (33.3%)	11 (40.7%)	17 (37.8%)	
Low	12 (66.7%)	16 (59.3%)	28 (62.2%)	

Chi-square correlation analysis of methylated (M) and unmethylated (U) *SFRP1* categories to different clinicopathological and experimental parameters

*m* male, *f* female, *DOD* died of disease, *NED* no evidence of disease, *HB* hepatoblastoma, *HCC* hepatocellular carcinoma, *TLCT* transitional liver cell tumor, *NSET* nested stromal-epithelial liver tumor, *E* embryonal, *F* fetal, *pure OS* pure osteoid, *C1 and C2* 16-gene signature cluster C1 and C2 (Cairo et al. 2008), *nd* no data, *na* not applicable

## Discussion

A constitutive activation of the WNT/β-catenin signaling pathway is a common event in pediatric liver tumor development. During embryogenesis, the pathway controls liver development and hepatoblast proliferation, indicating that a signaling malfunction contributes to liver cell transformation and tumor development (Perugorria et al. 2019). Indeed, HBs and HCCs exhibit an increased mutational burden in WNT/β-catenin pathway components that affect *CTNNB1*, *APC*, *AXIN1* and *AXIN2* genes (Tate et al. 2019). Although most of the mutations promote stabilization of β-catenin and pathway hyperactivation, several studies have reported that an additional epigenetic inhibition of WNT antagonists is important for cancer formation (Anastas and Moon 2013; Suzuki et al. 2008). In different tumor entities, an epigenetic silencing of various WNT antagonist, such as WIFs, DKKs and SFRPs, correlates with a poor prognosis or high-grade cancer (Kardum et al. 2017; Lin et al. 2017; Davaadorj et al. 2016). Interestingly, a restoration of the WNT antagonist expression attenuated tumor growth (Shih et al. 2007; Gumz et al. 2007). In the present study, we addressed the role of the WNT inhibitory factor *SFRP1* in HB and pediatric HCC, since its functional relevance has not been specified, yet. By determining the endogenous gene expression and DNA methylation status of the WNT antagonists *APC*, *DKK1*, *SFRP1* and *WIF1* in four HB cell lines, we detected a heterogeneous pattern. Although all four genes were methylated in HB cells, with the exception of *DKK1* in HuH-6 cells, only *APC* and *SFRP1* revealed a concomitant transcriptional repression. Moreover, a treatment of the tumor cells with 5-aza solely restored *SFRP1* expression in all four cell lines, indicating that DNA methylation is responsible for the *SFRP1* suppression in HB cell lines. We detected no involvement of histone acetylation on *SFRP1* regulatory gene sites. Nevertheless, besides DNA methylation, histone modifications might control the expression of other WNT antagonist. Based on these results, we focused our functional analyses on *SFRP1* and overexpressed the gene in the HB cell lines HuH6, HepT1 and HepG2. In line with studies in HCC and other tumor entities, a re-expression of *SFRP1* inhibited tumor cell growth, colony formation and migration of HB tumor cells (Shih et al. 2007; Kaur et al. 2012; Wang et al. 2018). In contrast to Shih et al. (2007), our data demonstrated that cell lines which possess β-catenin deletion mutations, such as HepT1 and HepG2 cells, also exhibited canonical WNT inhibition. Interestingly, *SFRP1* re-expression diminished the canonical WNT/β-catenin signaling activity and we detected a downregulation of the WNT target genes *MYC* and *CCND1,* although the HB cell lines carry beta-catenin mutations. Moreover, we uncovered that the overall level and cellular localization of β-catenin is not changed after restored *SFRP1* expression. Based on these results, we suppose that *SFRP1* expression abolishes β-catenin-driven transcription activity. In accordance, it was shown that the SFRP1-mediated inhibition of the WNT pathway was independent from wildtype or mutant β-catenin in colorectal cancer cells (Suzuki et al. 2004). Hence, the epigenetic silencing of the Wnt antagonists SFRP1 is an important mechanism in pediatric liver carcinogenesis, promoting WNT signaling-mediated oncogenic transformation.

Notably, in our pediatric liver cancer cohort, we detected a reduced *SFRP1* gene expression in around 62% (28/45) of cases. To our knowledge, we show for the first time that low *SFRP1* expression correlated significantly with a worse classification in the PRETEXT stratification system and to β-catenin mutations, indicating that *SFRP1* gene silencing in β-catenin mutant cancer leads to a more aggressive cancer growth. SFRP1 repression might potentiate the oncogenic function of the WNT signaling pathway, particularly in β-catenin mutant cancers. Thus, a restoration of the *SFRP1* expression through epigenetic drugs or the natural compound flavonoid epigallocatechin-3-gallate (EGCG), as it was shown in our recent study, could be a new therapeutic option for β-catenin mutant pediatric liver cancers (Godeke et al. 2013). In contrast to other studies, the reduced *SFRP1* gene expression in our HB patient cohort was not associated to the DNA methylation status (Vincent and Postovit 2017; Kaur et al. 2012). Several patient samples showed a *SFRP1* downregulation without a concomitant methylation of the promoter. With the help of the MSP, we determined the methylation status of only a few cytosines in the CpG island, thus we cannot exclude the possibility that adjacent cytosines might be methylated, which would result in gene silencing (Hernandez et al. 2013). Moreover, recent studies proposed a microRNA-dependent inhibition of *SFRP1* in different cancer entities, which may also occur in pediatric liver cancer (Ba et al. 2017; Li et al. 2017). Overall, we identified promoter methylation of *SFRP1* in 40% (18/45) of the analyzed cases. Importantly, the *SFRP1* methylation correlated with HCC-like pediatric liver tumors, with a higher age at diagnosis and with *TERT* mutations. It needs to be taken into consideration that these clinical features are dependent on each other, since the tumor manifests at older ages in the HCC/TLCT group (Tomlinson and Kappler 2012), which is furthermore characterized by frequent *TERT* mutations (Eichenmuller et al. 2014). Although in several tumor entities, *SFRP1* expression or methylation was proposed as a prognostic biomarker (Davaadorj et al. 2016; Zheng et al. 2015; Atschekzei et al. 2012), this was not the case for our cohort of pediatric liver cancers.

## Conclusion

In conclusion, we delineated an important role for the epigenetic silencing of *SFRP1* in pediatric liver cancer cell lines and patient samples. Our data demonstrated that β-catenin mutant pediatric liver cancers are accompanied by a suppression of the WNT antagonist *SFRP1*, which promotes malignant tumor cell characteristics. *SFRP1* methylation was highly associated to advanced pediatric liver tumors, with HCC-like features and *TERT* mutations. However, further studies are needed to clarify whether *SFRP1* gene expression or methylation could serve as a potential prognostic or diagnostic biomarker for HB or pediatric HCC.

## Electronic supplementary material

Below is the link to the electronic supplementary material.

Supplementary file1 (XLSX 20 kb)

Supplementary file2 (XLSX 11 kb)

Supplementary file3 (PDF 1363 kb)

Supplementary Figure 1: Histone acetylation has no impact on *SFRP1 *silencing in human HB cell lines.** a **Representative immunoblot images detecting SFRP1 protein levels after 72 h 1.5 µM 5-aza and 1.5 µM 5-aza/0.5 µM SAHA treatment of HuH-6, HepT1 and HepG2 cells. GAPDH served as loading control (*n *= 3). **b** ChIP analysis of H3K27ac at regulatory genome sites of *SFRP1* and *EPCAM* after 72 h 1.5 µM 5-aza and 1.5 µM 5-aza/0.5 µM SAHA treatment of HuH-6, HepT1 and HepG2 cells. ChIP DNA was quantified by qRT-PCR and normalized as percent of input (*n *= 2) file4 (PDF 151 kb)

Supplementary Figure 2: Re-expression of *SFRP1* abolishes WNT signaling activity**. a **mRNA expression of *SFRP1 *in transient pcDNA3.1- and pSFRP1-transfected HepT1 and HepG2 cells after 48 and 72 h was determined by qRT-PCR and calculated as normalized mRNA expression (fold change) to pcDNA3.1 control (*n *= 2). **b **Cell growth of transient pcDNA3.1- and pSFRP1-transfected HepT1 and HepG2 cells was assessed by MTT assay at indicated time points. Values were normalized to zero hour time points and shown as mean±SEM (*n *= 2). Slope difference was analyzed by linear regression, ****p < 0.0001. **c** Cell growth of stable pcDNA3.1- and pSFRP1-transfected HepT1 cells was assessed by MTT assay at indicated time points. Values were normalized to zero hour time point and shown as mean±SEM (*n *= 4). Slope difference was analyzed by linear regression, *p < 0.05. **d** Representative pictures and quantification of number of colonies per well of stable pcDNA3.1- and pSFRP1-transfected HepG2 cells (*n *= 3). **e** TOP/FOP reporter plasmid activity was assessed by a relative luciferase activity in stable pcDNA3.1- and pSFRP1-transfected HepT1 and HepG2 cells 48 h after co-transfection (*n *= 5) file5 (PDF 180 kb)

## Abbreviations

HB: Hepatoblastoma
HCC: Hepatocellular carcinoma
NL: Normal pediatric liver tissue
MSP: Methylation-specific-PCR
5-aza: 5-aza-2′-deoxycytidine
pSFRP1: PcDNA3.1-SFRP1 plasmid
TLCT: Transitional liver cell tumors
NSET: Nested stromal-epithelial liver tumors
PRETEXT: PRE-Treatment EXTent of tumor
EGCG: Epigallocatechin-3-gallate
m: Male
f: Female
DOD: Died of disease
NED: No evidence of disease
E: Embryonal
F: Fetal
pure OS: Pure osteoid
C1 and C2: 16-gene signature cluster 1 and 2 (Cairo et al. 2008)
M: Methylated
U: Unmethylated
nd: No data
na: Not applicable
