# Supplementary figures and images for: Downregulation of SFRP1 is a protumorigenic event in hepatoblastoma and correlates with beta-catenin mutations
Source: J Cancer Res Clin Oncol. 2020 Mar 18;146(5):1153–67. doi: 10.1007/s00432-020-03182-1 (PMC7142044; doi:10.1007/s00432-020-03182-1)

APC

control

3d

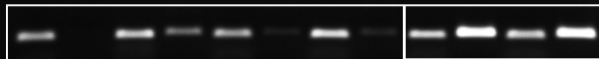

3d

5d

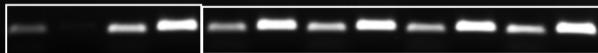

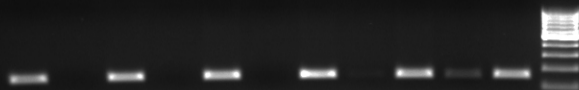

DKK1 - control

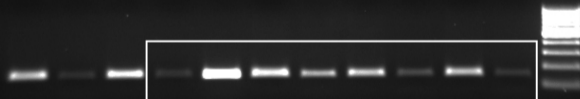

DKK 3d

DKK1 5d

SFRP1

control

3d

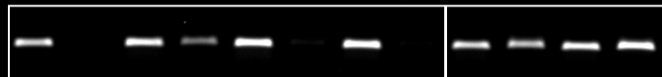

3d

5d

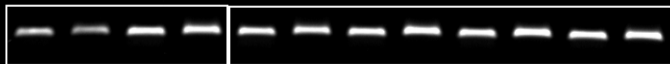

WIF1 3d

WIF1 control

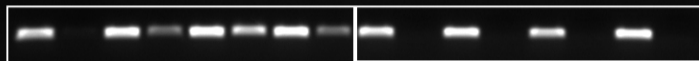

WIF1 5d

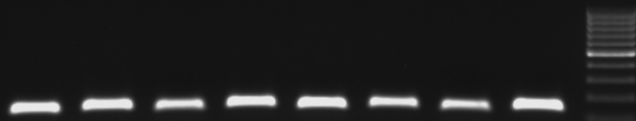

Sss1 5-Aza N110 N146

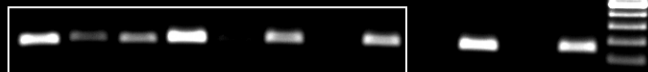

4

5

6

7

9

10

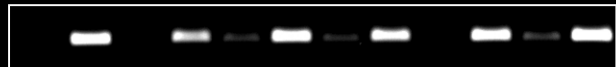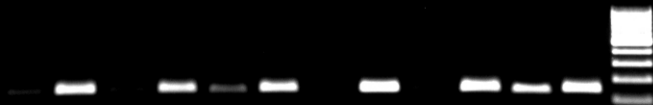

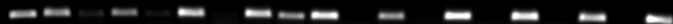

198 227 253 254

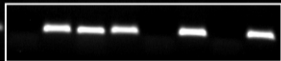

Supplement: Supplementary file 3 — Supplementary file3 (PDF 1363 kb) [file 432_2020_3182_MOESM3_ESM.pdf]

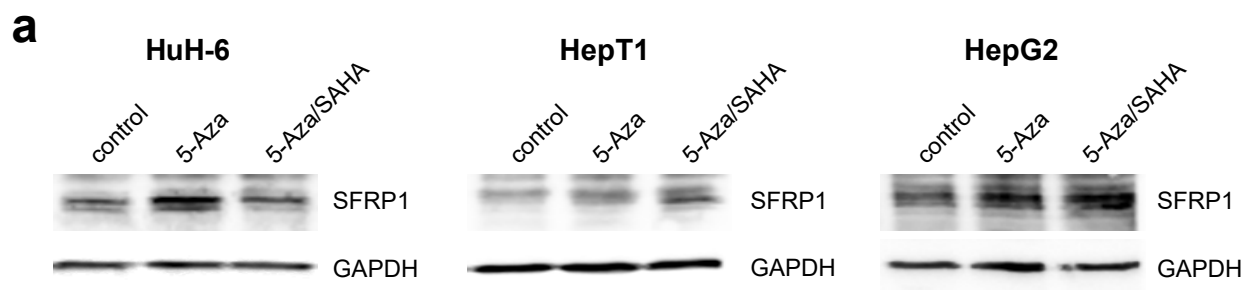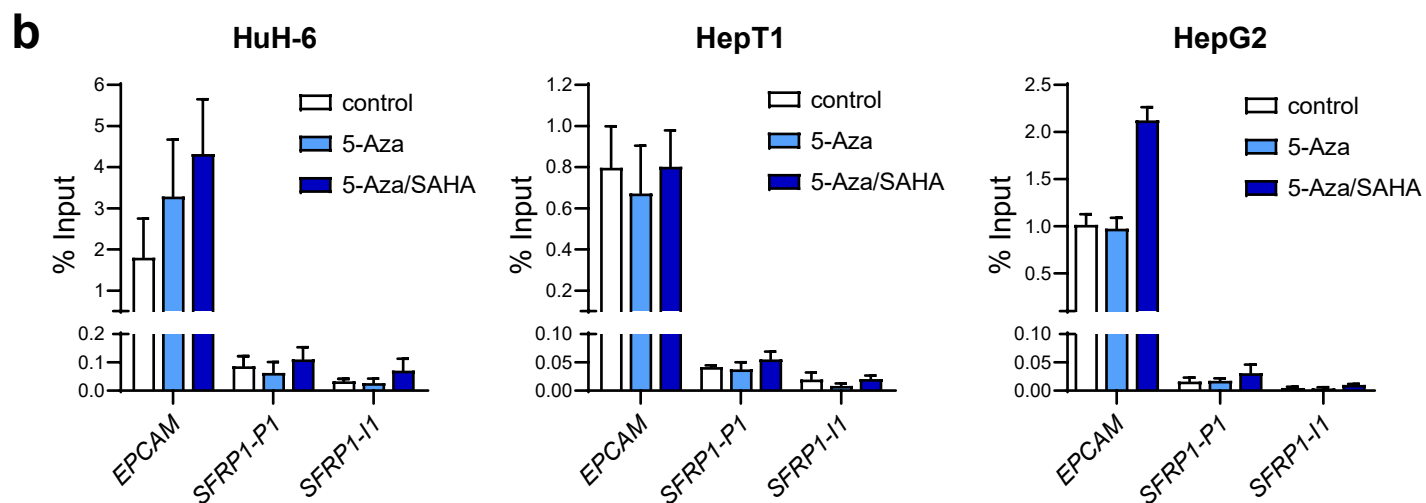

Supplement: Supplementary file 4 — Supplementary Figure 1: Histone acetylation has no impact on SFRP1 silencing in human HB cell lines. a Representative immunoblot images detecting SFRP1 protein levels after 72 h 1.5 µM 5-aza and 1.5 µM 5-aza/0.5 µM SAHA treatment of HuH-6, HepT1 and HepG2 cells. GAPDH served as loading control (n = 3). b ChIP analysis of H3K27ac at regulatory genome sites of SFRP1 and EPCAM after 72 h 1.5 µM 5-aza and 1.5 µM 5-aza/0.5 µM SAHA treatment of HuH-6, HepT1 and HepG2 cells. ChIP DNA was quantified by qRT-PCR and normalized as percent of input (n = 2) file4 (PDF 151 kb) [file 432_2020_3182_MOESM4_ESM.pdf]

**a**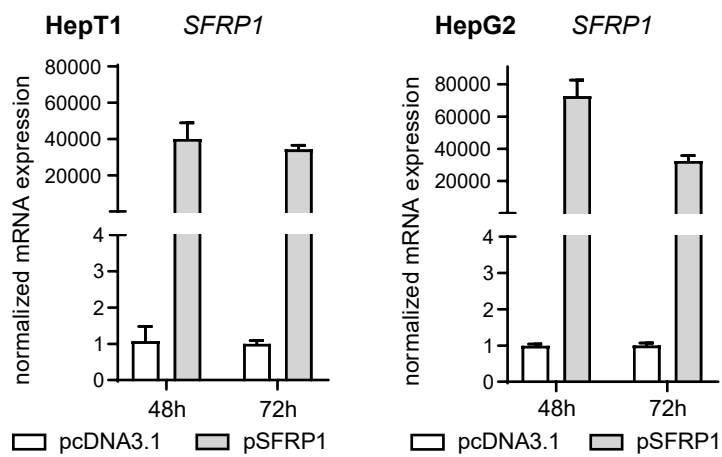**b**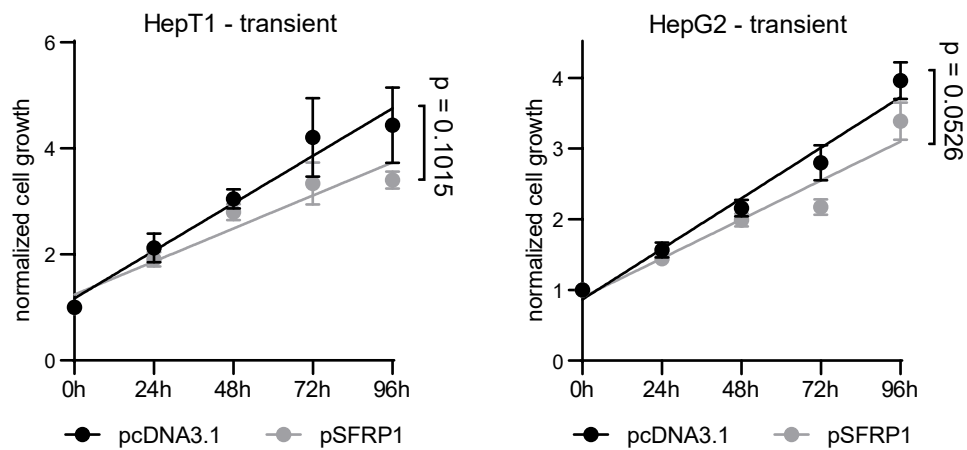**c**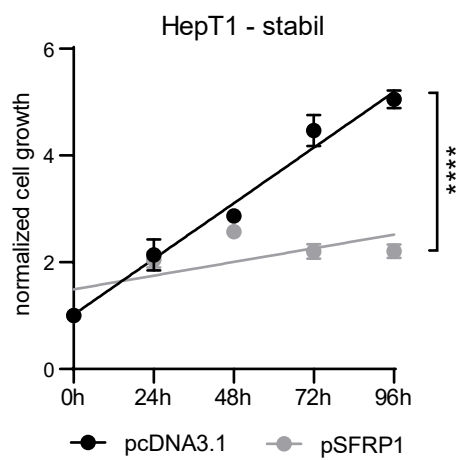**d**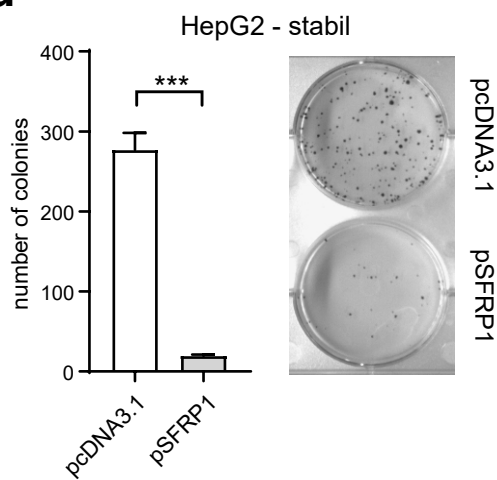**e**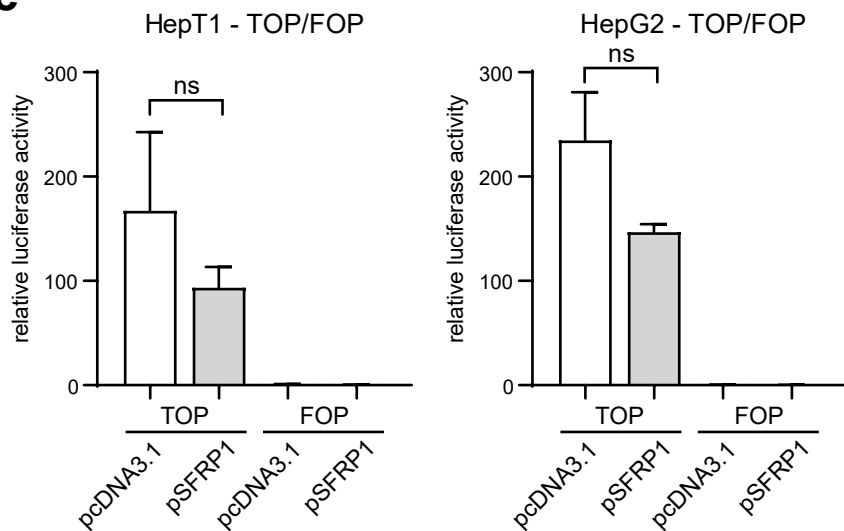

Supplement: Supplementary file 5 — Supplementary Figure 2: Re-expression of SFRP1 abolishes WNT signaling activity. a mRNA expression of SFRP1 in transient pcDNA3.1- and pSFRP1-transfected HepT1 and HepG2 cells after 48 and 72 h was determined by qRT-PCR and calculated as normalized mRNA expression (fold change) to pcDNA3.1 control (n = 2). b Cell growth of transient pcDNA3.1- and pSFRP1-transfected HepT1 and HepG2 cells was assessed by MTT assay at indicated time points. Values were normalized to zero hour time points and shown as mean±SEM (n = 2). Slope difference was analyzed by linear regression, ****p < 0.0001. c Cell growth of stable pcDNA3.1- and pSFRP1-transfected HepT1 cells was assessed by MTT assay at indicated time points. Values were normalized to zero hour time point and shown as mean±SEM (n = 4). Slope difference was analyzed by linear regression, *p < 0.05. d Representative pictures and quantification of number of colonies per well of stable pcDNA3.1- and pSFRP1-transfected HepG2 cells (n = 3). e TOP/FOP reporter plasmid activity was assessed by a relative luciferase activity in stable pcDNA3.1- and pSFRP1-transfected HepT1 and HepG2 cells 48 h after co-transfection (n = 5) file5 (PDF 180 kb) [file 432_2020_3182_MOESM5_ESM.pdf]
